# Hacker within! *Ehrlichia chaffeensis* Effector Driven Phagocyte Reprogramming Strategy

**DOI:** 10.3389/fcimb.2016.00058

**Published:** 2016-05-31

**Authors:** Taslima T. Lina, Tierra Farris, Tian Luo, Shubhajit Mitra, Bing Zhu, Jere W. McBride

**Affiliations:** ^1^Department of Pathology, University of Texas Medical BranchGalveston, TX, USA; ^2^Department of Microbiology and Immunology, University of Texas Medical BranchGalveston, TX, USA; ^3^Center for Biodefense and Emerging Infectious Diseases, University of Texas Medical BranchGalveston, TX, USA; ^4^Sealy Center for Vaccine Development, University of Texas Medical BranchGalveston, TX, USA; ^5^Institute for Human Infections and Immunity, University of Texas Medical BranchGalveston, TX, USA

**Keywords:** *Ehrlichia chaffeensis*, tandem repeat proteins (TRPs), cell signaling, post translational modification (PTM), innate immune response, secretion system, epigenetics

## Abstract

*Ehrlichia chaffeensis* is a small, gram negative, obligately intracellular bacterium that preferentially infects mononuclear phagocytes. It is the etiologic agent of human monocytotropic ehrlichiosis (HME), an emerging life-threatening tick-borne zoonosis. Mechanisms by which *E. chaffeensis* establishes intracellular infection, and avoids host defenses are not well understood, but involve functionally relevant host-pathogen interactions associated with tandem and ankyrin repeat effector proteins. In this review, we discuss the recent advances in our understanding of the molecular and cellular mechanisms that underlie *Ehrlichia* host cellular reprogramming strategies that enable intracellular survival.

## Introduction

*Ehrlichia* species are gram negative, obligately intracellular alpha-proteobacteria which belong to the family Anaplasmataceae in the order Rickettsiales (Anderson et al., 1991; Dumler et al., 2001). Anaplasmataceae includes the genera *Anaplasma, Neorickettsia*, and *Wolbachia*. The *Ehrlichia* genus contains six named species: *E. chaffeensis, E. ewingii, E. canis, E. muris, E. ruminantium*, and *E. ovis*, which infect a variety of vertebrate hosts (Buller et al., 1999; Olano et al., 2004; Allsopp et al., 2005; Perez et al., 2006). Two recently described novel *Ehrlichia* spp. have been identified, *Ixodes ovataus Ehrlichia* (IOE) and *E. muris-*like agent (EMLA) (Sotomayor et al., 2001; Pritt et al., 2011; Allen et al., 2014). *E. chaffeensis, E. ewingii*, and EMLA are considered human zoonotic pathogens, but *E. canis* infections in humans have been reported in South America. *E. chaffeensis, E. canis*, and *E. ewingii* naturally infect canids causing clinical manifestations ranging from poly arthritis to death (Breitschwerdt et al., 1998; Goldman et al., 1998; Dumler et al., 2001). Almost a century ago, *E. ruminantium* was isolated from cattle as the etiologic agent of heartwater (Cowdry, 1925). Shortly thereafter, *E. canis* was identified in the monocytes of tick-infected Algerian dogs (Donatien and Lestoquard, 1935). Over fifty years later, *Ehrlichia* infections in humans were first reported, and *E. chaffeensis* was subsequently identified in 1992 as an emerging zoonotic pathogen and the etiologic agent of human monocytotropic ehrlichiosis (HME) (Anderson et al., 1992). Most recently, infections with *E. ewingii* and EMLA have emerged in humans (Buller et al., 1999; Pritt et al., 2011; Allen et al., 2014).

HME is a group I NIAID, emerging, tick-borne zoonosis that manifests as sepsis or toxic shock syndrome. Patients exhibit flu-like symptoms that include fever, myalgia, malaise, and headache. Hematological abnormalities include leucopenia, anemia, thrombocytopenia, and elevated hepatic aminotransferases (Ismail et al., 2010). Although, more than 6000 cases have been reported to the Centers for Disease Control as of 2010, this number likely underestimates the actual number of cases by 100-fold based on estimates from prospective studies (Olano et al., 2003). HME is often underdiagnosed due to its non-specific symptoms, but is a serious disease that results in patient hospitalization in 43–62% of cases (Fishbein et al., 1994). Progression of the disease can result in multisystem failure, with adult respiratory distress syndrome (ARDS), meningitis, hepatic, and renal failure being common in many fatal cases (3%) (Paparone et al., 1995; Patel and Byrd, 1999). *E. chaffeensis*, which is transmitted through tick bite, is able to replicate in mammalian hosts and the tick vector (Ismail et al., 2010). The geographical distribution of *E. chaffeensis* infections coincides with the tick vector (*Amblyomma americanum*) and the white-tailed deer (*Odocoileus virginianus*), which serves as the primary reservoir in nature (Paddock et al., 1997).

Despite its small genome and limited number of effector proteins, *Ehrlichia* efficiently establishes an intracellular infection and avoids immune defenses in vertebrate and invertebrate hosts through complex molecular and cellular reprogramming strategies. Thus, *E. chaffeensis* is an excellent model organism to study host-pathogen interactions and to understand the molecular pathobiology of obligately intracellular microbes. This review will highlight the most recent advances in our knowledge of *Ehrlichia* molecular and cellular interactions, including the role newly described tandem repeat protein (TRPs) effectors play in exploiting host cell-signaling pathways, chromatin epigenetics, post-translational pathways, in order to subvert innate immune defenses.

## Physical characteristics and the genome

Individual ehrlichiae are coccoid to pleomorphic and vary in size from small (0.4 μm) to large (between 1 and ≤ 2 μm) (Popov et al., 1995). *E. chaffeensis* replicates in an intracellular, membrane-bound vacuole derived from host cell membrane, forming microcolonies called morula because they resembling mulberries. Morula is derived from the latin word “morum” for mulberry. Each vacuole contains one to more than 400 ehrlichiae (Barnewall et al., 1997). *E. chaffeensis* exhibits tropism for mononuclear phagocytes, and has a biphasic developmental cycle which involves two morphologically distinct forms, the smaller (0.4–0.6 μm), infectious dense cored cell (DC), and a larger replicating reticulate cell (RC, 0.7-0.9 μm). Ehrlichiae have a gram negative envelope which include a cytoplasmic membrane and outer membrane separated by periplasmic space; however, their cell wall lacks peptidoglycan (PG) (Mavromatis et al., 2006). DCs are usually coccoid in shape and characterized by an electron dense nucleoid that occupies most of the cytoplasm while RCs are pleomorphic in shape and have uniformly dispersed nucleoid filaments and ribosomes distributed throughout the cytoplasm (Zhang et al., 2007).

*E. chaffeensis* has one of the smallest bacterial genome (~1.3 Mb), encoding up to 1200 proteins, and about half of these genes have predicted or known functions. The genome sequence of *Ehrlichia* species has revealed low GC content (~30%), numerous long tandem repeat sequences (TRs) and one of the smallest genome to coding ratios, which is attributed to long non-coding regions (Dunning Hotopp et al., 2006; Frutos et al., 2006). Presence of long non coding regions and low GC content are thought to represent degraded genes in the final stage of elimination, and increased GC to AT mutations found in related Rickettsiales members (Andersson and Andersson, 1999a,b). TRs are actively created and deleted through an unknown mechanism that appears to be compatible with DNA slippage. Generation of TRs in *Ehrlichia* serves as a mechanism for adaptation to the hosts, not to generate diversity. Though TRs share similar characteristics, there is no phylogenetic relationship between the TRs from different species of *Ehrlichia*, suggesting TRs evolved after diversification of each species (Frutos et al., 2006).

The genome sequence of *Ehrlichia* has revealed a number of genes potentially involved in host-pathogen interactions including genes coding for tandem and ankyrin-repeat containing proteins, outer membrane proteins, actin polymerization proteins, and a group of poly(G-C) tract containing proteins, which may be involved in phase variation. Notably, genes encoding proteins associated with biosynthesis of peptidoglycan (PG) and lipopolysaccharide (LPS) are absent from the genome. Since, PG and LPS bind to nucleotide-binding oligomerization domain (Nod)-like receptor proteins and toll-like receptor proteins (TLR4) to activate leukocytes, the absence of LPS and PG presumably helps *Ehrlichia* to evade the innate immune response elicited by these pathogen-associated molecular patterns (PAMPs). *E. chaffeensis* contains two types of TRs, small (12 bp) and large (100–300 bp) period repeats. These TRs may play role in regulation of gene expression and phase variation (Frutos et al., 2007).

Multiple secretion systems have been described in gram negative bacteria for the delivery of effector proteins. In the ehrlichial genome, type I and IV secretion systems have been identified (Collins et al., 2005; Dunning Hotopp et al., 2006; Mavromatis et al., 2006). *E. chaffeensis* expresses three two-component systems (TCS), including histidine sensor kinases: CcKA, NtrY, and PleC and three response regulators, CtrA, NtrX, and PleD that contain conserved receiver domains with aspartate phosphorylation sites. These TCS are expressed sequentially during the life cycle of *Ehrlichia*, enabling detection and response to environmental signals by regulating gene expression (Cheng et al., 2006; Kumagai et al., 2006). *Ehrlichia* has decreased coding capacity for genes involved in transport and regulatory functions. ORFs encoding σ70 (rpoD) and σ32 (rpoH) are present but σ24 (rpoE) and σ54 (rpoN) are absent from the genome (Dunning Hotopp et al., 2006).

## Intracellular developmental biology

*E. chaffeensis* preferentially infects monocytes-macrophages and its intracellular life cycle is confined to membrane bound vacuoles. After entry through receptor-mediated endocytosis (1 h), the DC transition into an intermediate form (IM)-1, then into a replicating RC. RCs divide by binary fission for 48 h, and then transform into the second intermediate form (IM)-2, ending the cycle as fully mature DCs by 72 h post-infection (Zhang et al., 2007). DC ehrlichiae attach and enter the host cells by interacting with the surface protein DNaseX, and possibly other glycosylphosphatidylinositol (GPI)-anchored proteins associated with caveolae (Lin and Rikihisa, 2003b; Mohan Kumar et al., 2015). The ehrlichial proteins that serve as adhesins include TRP120 which is preferentially expressed by DC ehrlichiae, and the outer membrane invasin, entry-triggering protein or EtpE (ECH1038) (Popov et al., 2000; Mohan Kumar et al., 2013; Luo et al., 2015). The C-terminus of EtpE directly binds to mammalian protein DNaseX and facilitates *Ehrlichia* entry by interacting with CD147 and hnRNP-K and activating N-Wiskott-Aldrich syndrome protein (N-WASP) (Mohan Kumar et al., 2015). Recently, it has been determined that ehrlichial TRPs interact with an unknown receptors on the host cell surface activating canonical and noncanonical Wnt signaling pathways of the host, thereby stimulating phagocytosis and host cell entry (Luo et al., 2015). Others have demonstrated that a bacterial second messenger cyclic-di-GMP, and a serine protease HtrA expressed on *E. chaffeensis* surface regulates the stability of TRP120 and ehrlichial internalization (Kumagai et al., 2010). The phagosomes by which *E. chaffeensis* enters the host cells have characteristic features that include caveolin 1, G_M1_ ganglioside and phospholipase Cγ2 (Barnewall et al., 1997). Induction of receptor-mediated phagocytosis also triggers signaling events including transglutamination, tyrosine phosphorylation and activation of phospholipase Cγ2 (PLC-γ2), inositol-(1,4,5)-trisphosphate (IP_3_) production, and release of intracellular calcium (Lin et al., 2002; Lin and Rikihisa, 2003b). Recently, induction of these signaling events have been shown to be directly associated with TRP effectors and activation of canonical and noncanonical Wnt pathways (Luo et al., 2015).

**Figure 1:**
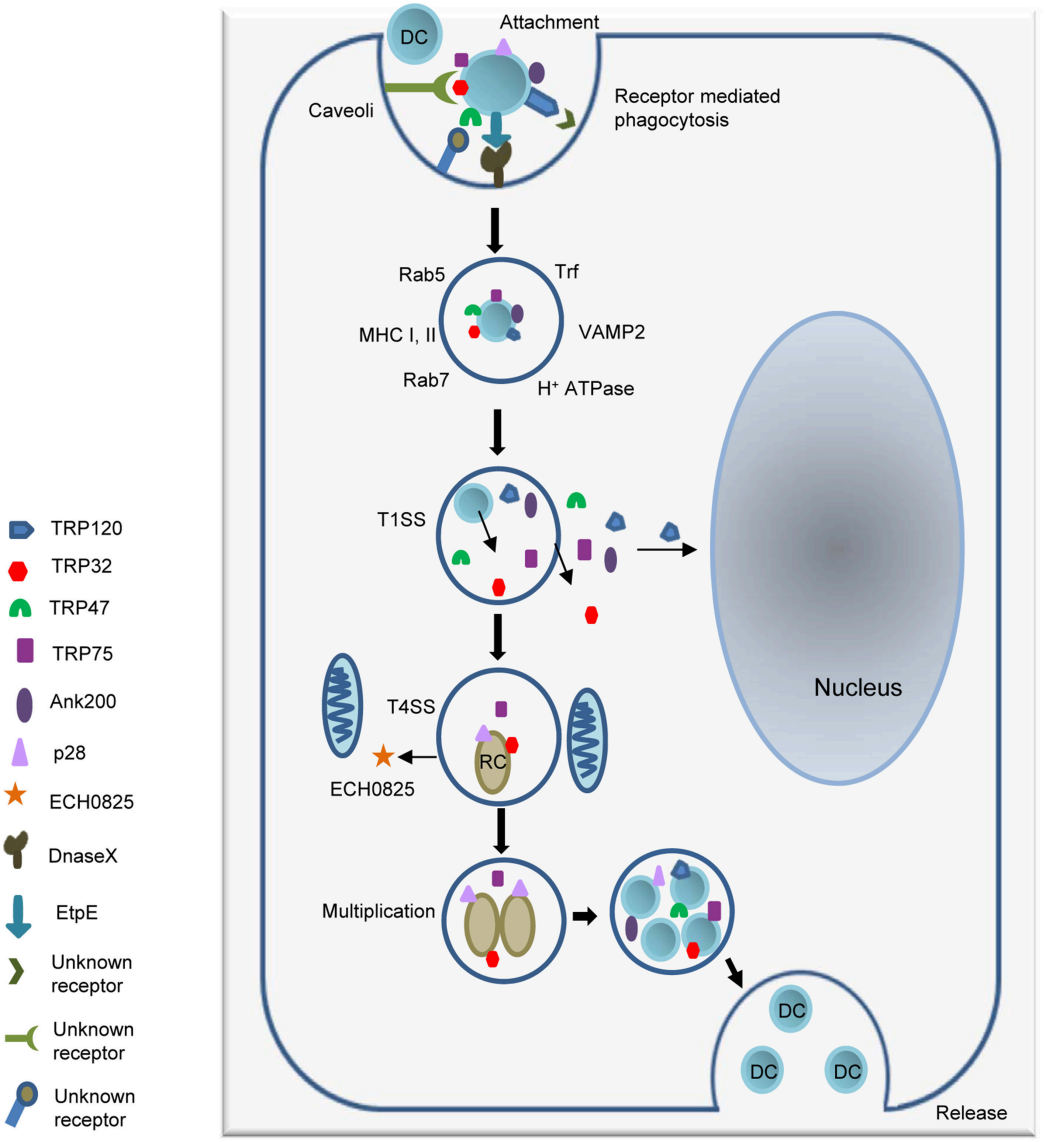
**Attachment and intracellular developmental cycle of *E. chaffeensis* in a mammalian host cell**. Infectious dense cored (DC) ehrlichiae that have well characterized surface proteins including TRP120, TRP47, and EtpE interact with host cell receptors such as the GPI anchored protein DNaseX and other unknown receptors, triggering receptor mediated phagocytosis. Once inside the host cell, DC ehrlichiae replicates in a membrane bound cytoplasmic vacuole and recruits both early and late endosomal proteins including Rab5, Rab7, and VAMP2 to the vacuole. The T1SS effector proteins TRP120, TRP32, TRP47, TRP75, and Ank200 are secreted into the intramorular space and translocate to host cytosol. TRP120 translocates to nucleus. DC ehrlichiae differentiate into replicating reticulate cells (RC) starting 1 h post infection and divide by binary fission every 8 h for next 48 h to form microcolonies known as morulae. The RC form secrets the T4SS effector ECH0825 and T1SS effectors TRP75 and TRP32. By 72 h post infection RC forms have transitioned back into infectious DC ehrlichiae. The ehrlichiae are released by exocytosis or cell lysis.

The ehrlichial cytoplasmic vacuole has features of early endosomes, such as the presence of Rab5, transferrin, transferrin receptor (TfR, CD71), early endosomal antigen 1 (EEA1), and vacuolar H^+^-ATPase. Some ehrlichial inclusions also contain major histocompatibility complex (MHC) class I and II, and vesicle associated membrane protein 2 (VAMP2) (Barnewall et al., 1997; Mott et al., 1999). Recently proteomic analysis detected late endosomal markers such as Rab7 along with Rab5, and TfR (Cheng et al., 2014). The ehrlichial vacuoles do not fuse with lysosomes, but the mechanisms behind inhibition of lysosomal fusion are still not clear and will require further investigation. *Ehrlichia* can be transported to neighboring cells through filopodia during initial stages of infection, or infectious DCs can be released by cell lysis to start a new infection cycle (Thomas et al., 2010; Figure 1).

## Secretion systems and effectors

Gram-negative bacteria secrete a variety of effectors and toxins through various secretion systems (1-6). *E. chaffeensis* has a type IV secretion system (T4SS) and type I secretion system (T1SS), but lacks a T3SS.

### Type I secretion system

The T1SS is widespread among gram-negative bacteria and is commonly used for the secretion of factors involved in nutrient acquisition and virulence. It is an ATP-binding cassette (ABC) transporter system consisting of an ATP-binding cassette protein (ABC, ECH0383), a membrane fusion protein of the HlyD family (MFP, ECH0970), and a TolC outer membrane protein (ECH1020). Together, these proteins create a channel which allows for one-step secretion of specific effectors from the bacterial cytoplasm to the extracellular environment. This secretion is dependent on recognition of a noncleaved signal present in the C-terminal 50 amino acids (AA). Although a conserved sequence has not been identified, T1SS substrates are typically repeat containing proteins with enrichment of [LDAVTSIF] AA and a paucity of [KHPMWC] AA in the 50 AA C-terminal region of the protein (Delepelaire, 2004). Using a heterologous type 1 secretion apparatus of *Escherichia coli* several *E. chaffeensis* T1SS substrates have been experimentally identified, including the 200 kDa ankyrin repeat protein (Ank200) as well as several tandem repeat proteins (TRPs) that have features similar to other type 1 secretion system substrates such as the repeats in toxin (RTX) family (Wakeel et al., 2011). Although studies to confirm secretion of TRPs by *E. chaffeensis* T1SS have not been performed, secreted TRPs have been detected in infected cells and cell culture supernatant, suggesting that are indeed T1SS substrates.

### Type IV secretion system

The T4SS is a nearly ubiquitous transport system found in a variety of both gram-positive and gram-negative bacteria. The archetypal gram-negative T4SS occurs in *Agrobacteria tumefaciens* and consists of 12 proteins (VirB1-11 and VirD4) organized into two loci that form a translocating pore complex and ATPase motor for energy dependent export of DNA and proteins (Christie et al., 2014). *E. chaffeensis* contains genes coding for VirB and VirD proteins. Interestingly, *E. chaffeensis* contains multiple duplications including four non-identical versions of VirB4 (ATPase) and VirB6 (inner membrane channel component) separated into five loci. Additionally, all VirB6 homologs were 3–10-fold larger than the prototypical *A. tumefaciens* VirB6. All components are co-expressed and interact during infection, suggesting that *E. chaffeensis* may possess a structurally novel inner membrane translocon (Cheng et al., 2008; Bao et al., 2009; Rikihisa et al., 2009). The *E. chaffeensis* T4SS is upregulated during the exponential growth phase in the monocyte and is also expressed in tick cells, supporting the idea that this system may play an important role in *E. chaffeensis* growth and virulence. Although several hypothetical T4SS substrates have been identified in *E. chaffeensis* including ECH0261, ECH0767, ECH0389, ECH0653, ECH0684, ECH0877, and ECH0825, only one T4SS substrate (ECH0825) has been experimentally confirmed. ECH0825 interacts with VirD4 and is secreted during infection, where it localizes to the host cell mitochondria and can inhibit host cell apoptosis (Liu et al., 2012).

### Characteristics of *E. chaffeensis* TRP and Anks

**Figure 2:**
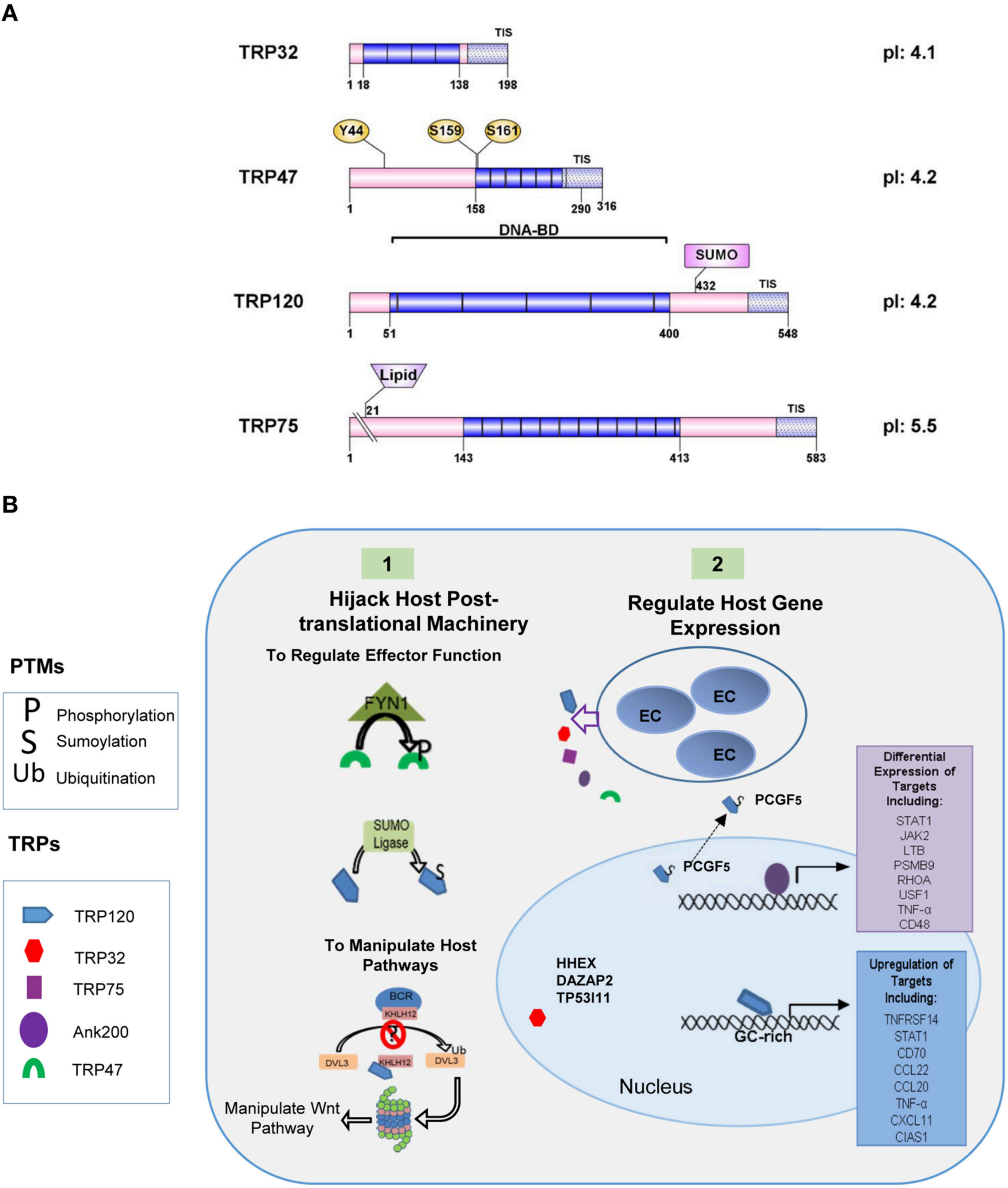
**Illustration of TRP effector domains. (A)** TRPs are a post-translationally modified effectors. Many modifications have been detected in the tandem repeat domains which also have been shown to contain the DNA-binding domain. SUMOylation sites (SUMO) are identified by pink rectangles. **(B)** *E. chaffeensis* effectors subvert host cellular functions. **(1)** Ehrlichial effectors hijack host post-translational machinery and acquire post-translational modifications that regulate effector function and interactions. TRP47 interacts with the tyrosine kinase FYN1 and is phosphorylated. TRP120 is SUMOylated by SUMO ligase UBC9 and may involve other undefined SUMO E3 ligase. This PTM is required for multiple interactions with SUMO-binding domain containing host proteins such as PCGF-5 and GGA1. *Ehrlichia* effectors also interact with host PTM machinery to manipulate host pathways. TRP120 interacts with Kelch-like 12 (KLHL12) an E3 ligase in the BCR (BTB-CUL3-RBX1) complex which ubiquitinates disheveled 3 (DVL3) in order to negatively regulate Wnt signaling. TRP120 interaction with KLHL12 may prevent this. **(2)** Ehrlichial effectors also regulate host gene expression. SUMOylated TRP120 interacts with PCGF5 and resulting in recruitment out of the nucleus. Ank200 and TRP120 directly interact with host chromatin leading to the differential expression of host genes. TRP32 interacts with several host transcription factors and may also influence host gene expression.

Many TRPs have been molecularly characterized, initially as antigens that elicit strong protective antibody responses during infection directed at continuous species-specific epitopes located in the TR region (Doyle et al., 2006; Luo et al., 2008, 2009; Kuriakose et al., 2012). The TR domains in TRP32, TRP47, and TRP120 are serine-rich and acidic while the TRP75 TR domain is lysine-rich and basic (Luo et al., 2008, 2009, 2010, 2011; McBride et al., 2011). Despite these similarities, the TRs found in each protein possess distinct AA sequences that vary both in length, and number. Additionally, the number of repeats differs between strains, with the greatest variability observed in TRP32, which has between 3 (Sapulpa isolate) and 6 (Wakulla isolate) repeats (Buller et al., 1999). The TRPs range from 198 AAs (TRP32) to 583 AAs (TRP75) in length, but all migrate at a higher molecular mass than predicted from their sequences due to their acidic properties (Luo et al., 2009; McBride et al., 2011). TRP32, TRP75, and TRP120 possess relatively large C-terminal domains, while TRP47 has a small C-terminus (26 AAs). Despite these differences T1S signals were identified in the C-terminal domains of all of the TRPs (Wakeel et al., 2011). TRP32 and TRP75 are constitutively expressed by both DCs and RCs, while TRP47 and TRP120 are expressed by DCs only (Popov et al., 2000; Doyle et al., 2006; Luo et al., 2008; McBride et al., 2011). All TRPs are transcriptionally active in tick cells, but only TRP120 was detected at the protein level (Kuriakose et al., 2011). TRPs are modified by multiple host-mediated post-translational modification pathways, including phosphorylation and ubiquitination/SUMOylation and localize to various subcellular locations, including the nucleus (Figure 2A) (Huang et al., 2008; Wakeel et al., 2010; McBride et al., 2011; Zhu et al., 2011; Dunphy et al., 2014). The most extensively studied ankyrin-repeat protein in *E. chaffeensis* is Ank200, a major immunoreactive protein and an effector protein that has a central region containing multiple ankyrin repeats flanked by acidic N- and C-terminal regions containing major linear antibody epitopes (Luo et al., 2010). Ank200 is also secreted by T1SS and translocates to the host nucleus (Zhu et al., 2009; Wakeel et al., 2011).

## Modulation of host gene expression

During *E. chaffeensis* infection, the host transcriptome exhibits differential expression of 5–10% of host genes (McBride and Walker, 2011). Host gene expression appears to be modulated in part by three primary pathogen directed modi operandi: direct regulation of host gene expression by ehrlichial nucleomodulins, modulation of host epigenetic marks, and activation of host cell signaling pathways that act as nexuses in cell decision-making processes. Direct transcriptional regulation represents an efficient means of targeting these cell-fate nexuses. Transcription factors can regulate the expression of hundreds to thousands of gene targets while epigenetic regulators can have an even broader impact on cell fate. The first *Ehrlichia* nucleomodulin described was Ank200, which binds to repetitive AT-rich regions called *Alu* elements within the promoters and intergenic regions of genes involved in transcriptional regulation, ATPase activity, and apoptosis regulation (Zhu et al., 2009). Ank200 targets are differentially regulated during infection with the majority being downregulated, but some being highly upregulated. This is similar to *Anaplasma phagocytophilum* (*A. phagocytophilum*) AnkA, which also binds AT-rich regions within the promoters of target genes and is able to significantly decrease expression of its target genes. AnkA gene repression occurs concurrently with a decrease in acetylation of proximal histones, which suggests an epigenetic mechanism is involved (Garcia-Garcia et al., 2009). *E. chaffeensis* Ank200 might also function by binding specific genes and recruiting host epigenetic regulators to repress expression of target genes.

Interactions between multiple ehrlichial nucleomodulins may be necessary for regulating gene expression, as well as temporal regulation of gene expression by individual TRPs. TRP120 binds DNA via a tandem repeat DNA binding domain, which is similar to that described in the transcription activator-like (TAL) effectors *of Xanthomonas* and *Ralstonia* sp. TRP120 binds a GC-rich motif and targets genes involved with transcriptional regulation, signal transduction, and apoptosis (Figure 2B). TRP120 target genes were significantly upregulated during infection and this phenotype was duplicated when TRP120 protein was transfected into cells (Zhu et al., 2011).

## TRP-host protein interactions

Recently numerous novel *Ehrlichia*-host protein interactions have been identified using a yeast two-hybrid (Y2H) approach, which has helped define the complex mechanisms by which *E. chaffeensis* modulates host cell processes (Wakeel et al., 2009; Luo et al., 2011; Luo and McBride, 2012). Multiple studies have determined that TRPs interact with a diverse network of host proteins involved in many host cellular processes including cell signaling, transcriptional and translational regulation, post-translational modification, intracellular trafficking, cytoskeletal organization, and apoptosis. Co-tranfection, co-immunoprecipitation and co-localization assays confirmed the interactions of each TRP with select host proteins during ectopic expression or during *Ehrlichia* infection. RNA interference assays have also confirmed the importance of these host proteins on ehrlichial survival.

Y2H results have also identified numerous putative common interacting host proteins of TRPs, including EF1A1, IGHA1, IGLL5 (interacting with both TRP32 and TRP120), PCGF5, IgKC, RP4, RPL11, CA1, CLC, and UBB (with TRP47 and TRP120), indicating the importance of overlapping targets and the crosstalk/convergence of defined cellular networks by *Ehrlichia* through its effectors (Wakeel et al., 2009; Luo et al., 2011; Luo and McBride, 2012). Notably, elongation factor 1 alpha (EF1A) is the second most abundant protein in eukaryotes after actin and is also one of the most important multifunctional eukaryotic proteins. In addition to its recognized primary role in translation, EF1A functions also include cytoskeletal remodeling, enzyme regulation, and apoptosis, (Condeelis, 1995; Ejiri, 2002). Polycomb group ring finger protein 5 (PCGF5) is a component of the polycomb repressive complex (PRC) which mediates epigenetic regulation (Junco et al., 2013). RPL11 is a subunit of 60 s ribosomal protein and is also involved in ribosomal entry and p53 mediated apoptosis (Donati and Thomas, 2012). The TRP interactions with a wide variety of regions of human immunoglobulins, suggest the association of TRPs with the host immune system or apoptosis (Yang et al., 2009).

TRP-interacting proteins also include host transcription factors. TRP32 interacts with DAZ-associated protein 2 (DAZAP2), a transcription factor associated with the canonical Wnt pathway, hematopoietically expressed homeobox (HHEX) which is required for hematopoietic cell differentiation, and elongation factor 1 alpha 1 (EF1A1), which is a component of transcription factor complex of T helper 1 cells (Maruyama et al., 2007; Lukas et al., 2009; Goodings et al., 2015). In addition to PCGF5, TRP120-interacting transcription factors include interleukin enhancer binding factor 3 (ILF3), a subunit of the nuclear factor of activated T-cells (NFAT), which is a transcription factor required for T-cell protein expression (Nakadai et al., 2015); lysine (K)-specific demethylase 6B (KDM6B) and transducin-like enhancer protein 4 (TLE4), both involved in regulation of transcription from RNA polymerase II promoter (Milili et al., 2002; Ohtani et al., 2013); nuclear receptor binding SET domain protein 1 (NSD1), which is a basic transcriptional factor and bifunctional transcriptional regulator (Lucio-Eterovic et al., 2010); and tripartite motif containing 24 (TRIM24), which mediates transcriptional control by interacting with the activation function 2 region of several nuclear receptors (Thenot et al., 1997). Therefore, irrespective of their role as direct transcriptional regulators, TRPs may regulate host gene transcription via their interactions with other host transcription factors and cofactors.

Notably, TRP Y2H studies have also discovered likely interactions with several apoptosis-associated proteins of the host cell (Luo et al., 2011). Several common interacting proteins of the TRPs, including EF1A1, RPL11 and various immunoglobulin light chains, which have been linked to apoptosis regulation. In addition, TRP32 interacts with tumor protein p53 inducible protein 11 (TP53I11), which is a downstream target of p53 and is also involved in the regulation of apoptosis (Wu et al., 2009). TRP47 interacts with adenylate cyclase-associated protein 1 (CAP1), which has been implicated in promoting apoptosis by functioning as an actin shuttle to mitochondria (Wang et al., 2008). TRP120 interacts with intercellular adhesion molecule 3 (ICAM3) (Luo et al., 2011), which can activate the Akt/ERK/CREB2 pathway and block apoptosis, and protein phosphatase 3 regulatory subunit B alpha (PPP3R1), which positively regulates protein insertion into the mitochondrial membrane and the apoptotic signaling pathway (Su et al., 2012; Ahn et al., 2013). Thus, the interactions of the TRPs with different host proteins associated with apoptosis may serve to inhibit or to promote apoptosis at different stages of infection to facilitate ehrlichial survival and release, respectively.

Interestingly, *E. chaffeensis* TRPs also interact with numerous host proteins associated with cellular signaling pathways, particularly Wnt pathways (Table 1). TRP32-interacting protein DAZAP2 is a highly conserved protein that modulates gene transcription driven by the Wnt/β-catenin signaling effector TCF (Lukas et al., 2009). TRP47-interacting protein CYP4F3 is a member of the cytochrome P450 superfamily of monooxygenases and is a target of Wnt/β-catenin pathway (Corcos et al., 2012). Many TRP120-interacting host proteins are involved in both canonical and noncanonical Wnt signaling pathways and Wnt ligand secretion. *E. chaffeensis* has recently been demonstrated to exploit Wnt pathways through TRP-Wnt signaling protein interactions (Luo et al., 2015). In addition, TRP120 interacts with ADAM17 metalloprotease, indicating that Notch signaling pathway may also be involved in the ehrlichial infection (Luo et al., 2011).

**Table 1:** ***E. chaffeensis* TRPs interact with host Wnt pathway associated components**.

**TRP**	**Host protein**	**Property/Function in Wnt signaling**
TRP32	DAZAP2	Modulates gene transcription driven by Wnt/β-catenin signaling effector TCF
TRP47	CYP4F3	A target of Wnt/β-catenin pathway
TRP120	ARID1B	Interacts with β-catenin to suppress Wnt signaling
	CEP164	Interacts with DVL3
	KDM6B	β-catenin binding; activates Wnt3 or DKK1 to stimulate or suppress Wnt signaling at different stages
	KLHL12	Interacts with DVL3 for degradation
	ILF3	A subunit of transcription factor NFAT
	LMO2	Interacts with Axin1 and DAZAP2
	IRF2BP2	Interacts with NFATC2 to repress transcriptional activity
	PPP3R1	Calcineurin regulatory subunit 1; calcium ion and calmodulin binding; calcium-dependent protein phosphatase activity; NFAT import into nucleus
	TLE4	Represses gene transcription by TCF
	VPS29	Retrograde transport of proteins from endosomes to the trans-Golgi network; Wnt ligand biogenesis, secretion, trafficking

## Post translational modifications

Protein post-translational modifications (PTMs), such as phosphorylation, acetylation, ubiquitination and SUMOylation regulate many cellular processes. PTMs are rapid, reversible, controlled and highly specific, and provide a tool to regulate protein stability, activity, and localization. Numerous examples exist where pathogens target, manipulate and exploit host PTMs to facilitate a survival strategy (Ribet and Cossart, 2010a). It is established that bacterial pathogens exploit host PTM machinery to promote bacterial survival and replication. Many bacterial effectors mimic host proteins involved in the host post-translational machinery to modify host proteins and signaling (Ribet and Cossart, 2010b).

### Phosphorylation

Protein phosphorylation plays a role in several key steps of the infectious process of bacterial pathogens such as adhesion to the host, triggering, and regulating pathogenic functions, altering host signaling cascades and impairing host defense mechanisms. The bacterial outer membrane is the primary contact between *Ehrlichia* and the host cell. The surface-exposed proteins in *E. chaffeensis* include the outer membrane protein family (OMP-1) (Ohashi et al., 2001) and secreted effectors TRP47 and TRP120 (Doyle et al., 2006; Luo et al., 2009, 2010; Wakeel et al., 2010). The differentially expressed OMPs are post-translationally modified by phosphorylation and glycosylation to generate multiple expressed forms (Singu et al., 2005). However, it is not clear how these PTMs affect protein function or interactions with the host cell. The TRPs exhibit high serine/threonine content and contain predicted sites for phosphorylation. TRP47 interacts with the Src family tyrosine kinase, Fyn, a key component of the αβTCR-coupled signaling pathway, which may be involved in the tyrosine phosphorylation of TRP47 (Wakeel et al., 2010). TRP75 and Ank200 are also tyrosine phosphorylated, although the specific modified residues remain undefined (McBride et al., 2011). It is not clear which protein kinases phosphorylate Ank200 or how this phosphorylation is regulated, but AnkA of *A. phagocytophilum* is tyrosine phosphorylated by the Abl-1 tyrosine kinase. However, there are some functional similarities between Ank200 and AnkA associated with host gene transcription (Garcia-Garcia et al., 2009; Zhu et al., 2009).

### SUMOylation

SUMOylation, the covalent attachment of a member of the small ubiquitin-like modifier (SUMO) family of proteins to lysine residues in targeted proteins, is an essential post-translational protein modification for all eukaryotic cells. A number of bacterial pathogens are known to directly target the SUMOylation system in order to modulate overall SUMOylation levels in the host cell (Ribet and Cossart, 2010c). However, intracellular bacteria that exploit host cell SUMOylation to modify pathogen proteins as part of their intracellular survival strategy has been limited to *Ehrlichia* and *Anaplasma* (Dunphy et al., 2014; Beyer et al., 2015). Recently, the *E. chaffeensis* T1S effector TRP120 was found to be modified by SUMO at a canonical consensus SUMO conjugation motif located in the C-terminal domain *in vitro*. SUMOylation site was further confirmed using a high-density microfluidic peptide array (Zhu et al., 2016). In human cells, TRP120 conjugation with SUMO2/3 isoforms mediates interactions with host protein targets such as polycomb repressive proteins, actin and myosin cytoskeleton components or GGA1, which is involved in vesicular trafficking. Inhibition of the host SUMO pathway with a small-molecule inhibitor significantly decreases interaction between TRP120 and PCGF5, as well as decreasing PCGF5 recruitment to the ehrlichial vacuole. More importantly, inhibition of this pathway also decreases ehrlichial intracellular survival (Dunphy et al., 2014).

### Ubiquitination

Another highly dynamic PTM that is implicated in signaling pathways is ubiquitination. Ub (ubiquitin) is a small, 76-amino acid protein which is highly conserved and widely expressed in all eukaryotic cells. Ubiquitination involves one or more covalent additions of Ub to the lysine residues of target proteins (i.e., mono- or poly-ubiquitination). Ubiquitin-dependent post-translational modification systems have important roles in several aspects of bacterial pathogenesis as well as in host defense responses. TRP120 was previously reported to interact with components of the Ub PTM pathways, including the E3 ligases, KLHL12, and FBW7 (F-box and WD repeat domain-containing 7, part of SCF, which is a E3 ligase complex), as well as Ub isoforms UBB and UBC, which suggests TRP120 is a target of Ub conjugation (Luo et al., 2011). However, it remains unclear whether the functional consequences of TRP120 ubiquitination are different from those associated with SUMOylation of TRP120. Thus, further study is needed to understand how Ub PTMs influence TRP120 function.

## Cytoskeletal organization and vesicle trafficking

Decreased expression of genes such as SNAP23 (synaptosomal-associated protein, 23 kDa), Rab5A (member of RAS oncogene family), and STX16 (syntaxin 16), which are involved in membrane trafficking are observed during *E. chaffeensis* infection. TRP120 and Ank200 bind genes involved in vesicle trafficking and cytoskeletal rearrangement such as clathrin (CTLA), syntaxins (SNX14, SNX11, SNX17), coatomer (COPA), and TSNARE1. At the protein level, TRP120 interacts with host proteins actin gamma 1 (ACTG1), actin related protein 2/3 complex (ARPC2), and unc-13 homolog D (UNC13D) (Luo et al., 2011). Since, inhibition of actin polymerization in *E. chaffeensis* infected cells prevents filopodia formation (Thomas et al., 2010), it is likely that the interaction of TRP120 with actins might play important role in ehrlichial entry and release from host cell. TRP47 interacts with CAP1 (actin binding protein adenylate cyclase protein 1) at the morula membrane interface and changes the distribution of CAP1 during infection. This multifunctional protein binds with actin, cofilin, SH3 domain, profilin, and adenylyl cyclase and is involved in receptor-mediated endocytosis and vesicle trafficking (Wakeel et al., 2009). It is possible that *Ehrlichia* mediated regulation of genes and protein expression associated with cytoskeletal components might facilitate vesicular trafficking, entry, and exocytosis during infection.

## Exploiting conserved cell signaling pathways

*E. chaffeensis* manipulates host cellular processes to create a favorable environment by reprogramming cell-signaling pathways and inhibiting bactericidal activity, most likely through specific interactions of its surface-expressed and/or secreted effector proteins. Intracellular survival and proliferation of *E. chaffeensis* involves activation of conserved cell signaling pathways (e.g., Wnt), suppression of tyrosine and mitogen-activated protein kinase (MAPK) activity and downregulation of Toll-like receptors and transcription factors, in monocytes and macrophages. Different gene targets of Ank200 and TRP120 are transcription factors in various host cell signaling pathways. Additionally, several host cell signaling proteins are regulated by TRPs and Ank200 at gene and protein levels (Zhu et al., 2009, 2011).

### Wnt signaling

**Figure 3:**
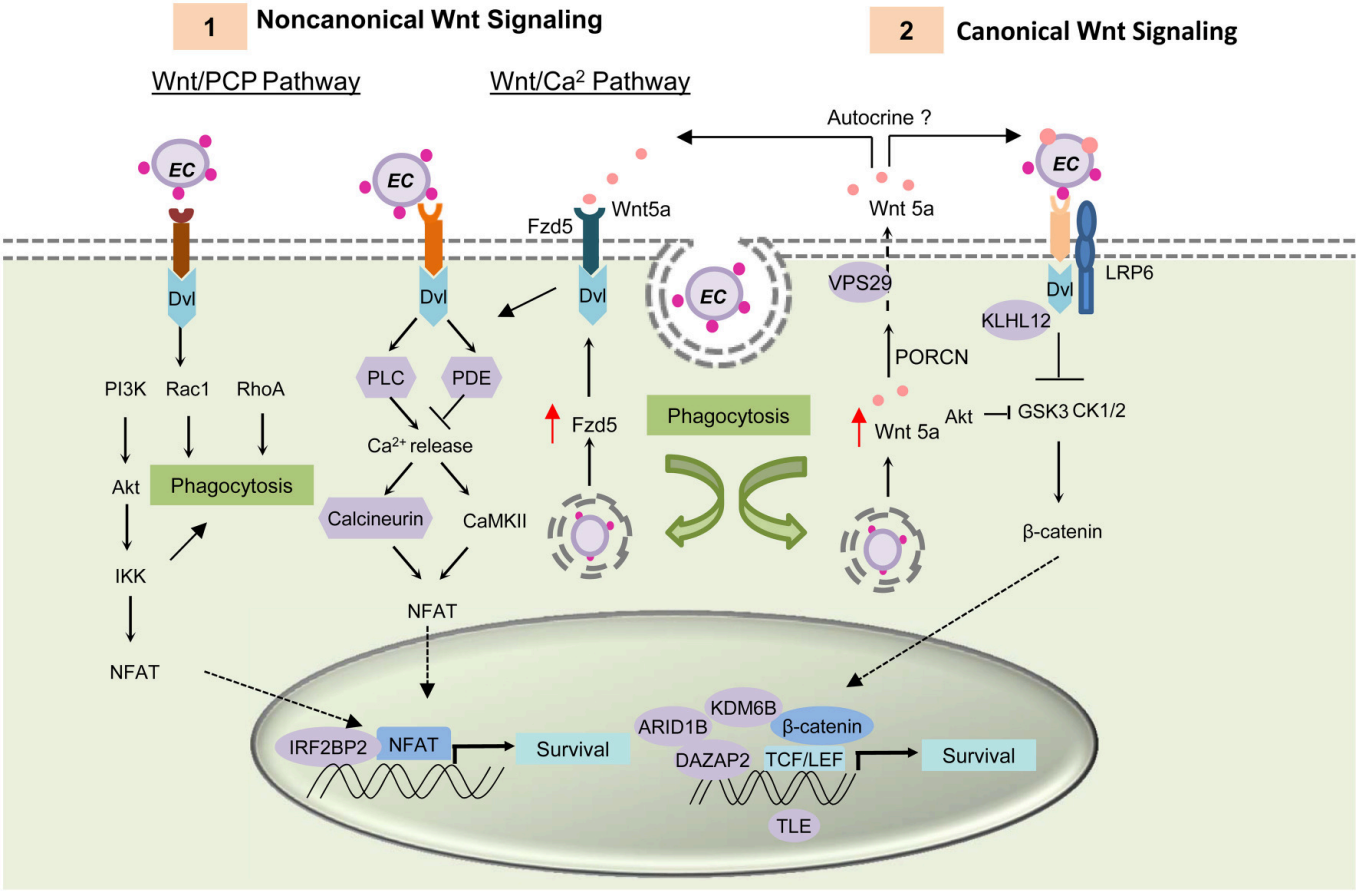
***E. chaffeensis* mediated activation of Wnt signaling pathway and function**. TRP proteins interacts with unknown Wnt receptors and activating both canonical and noncanonical Wnt signaling through activation of Dvl. **(1)** Activation of the Wnt/PCP pathway and the Wnt/ Ca^2+^ pathway causes translocation of transcription factor NFAT to the nucleus and results in target gene expression. TRP induced activation of noncanonical Wnt pathway activation triggers phagocytosis and helps in bacterial internalization. After internalization, *E. chaffeensis* induces expression of the receptor Fzd5 and possibly the ligand Wnt5a. Interaction of Wnt5a with Wnt receptor Fzd5 causes increased Ca^2+^ release and NFAT translocation to nucleus. This signaling plays a major role in ehrlichial survival. **(2)** Both ehrlichial TRPs and Wnt5a can interact with the unknown receptor and LRP6 co-receptor and activate canonical Wnt signaling pathway. Activation of canonical Wnt signaling results in dephosphorylation and translocation of β-catenin into the nucleus within 1 h p.i. Unphosphorylated β-catenin associates with TCF/LEF family of transcription factors and causes induction of Wnt target genes. Activation of these genes are essential for ehrlichial survival. TRPs interact with important components and regulators of Wnt pathway (shown in purple) and thus regulate Wnt signaling.

Previously, Wnt pathway components and regulators were found to interact with ehrlichial TRP effectors (Table 1) (Luo et al., 2011). Some of these interactions need further confirmation in mammalian cells; however, exploitation of the Wnt pathway by *E. chaffeensis* has been conclusively established. Most recently, it was demonstrated that host Wnt signaling plays an important role in ehrlichial internalization and infection, and that ehrlichial TRPs mediate bacterial invasion and survival through activation and modulation of Wnt signaling pathways (Luo et al., 2015). Canonical and noncanonical Wnt signaling is significantly stimulated during early stages of infection (1–3 h), as expression of Wnt signaling genes are altered, which coincides with dephosphorylation and nuclear translocation of β-catenin and NFATC1. Knockdown of major Wnt signaling molecules such as Wnt5a, Fzd5, β-catenin and NFAT, or TRP-interacting Wnt pathway components/regulators such as ARID1B, KDM6B, IRF2BP2, PPP3R1, and VPS29, results in significant reductions in ehrlichial load. Wnt5a-Fzd5 signaling appears to be very important for *Ehrlichia* survival after internalization, consistent with previous report that Wnt5a-Fzd5 signaling reduced bacterial killing by macrophages (Maiti et al., 2012). Moreover, small molecule inhibitors specific for canonical and noncanonical Wnt pathways components and Wnt ligand secretion significantly decrease ehrlichial load (Figure 3; Luo et al., 2015).

TRPs directly activate Wnt signaling and trigger phagocytosis (Luo et al., 2015). TRP-induced phagocytosis appears to be primarily a noncanonical mode of Wnt signaling most likely through Rac1-PI3K-IKK of Wnt/PCP signaling, similar to Wnt5a-induced phagocytosis; however it appears that *Ehrlichia* internalization is dependent on TRP/receptor interaction and independent of Wnt ligand secretion. Further investigation is needed to identify the TRP-interacting receptor and understand the importance of specific Wnt pathways in ehrlichial pathobiology.

### Notch signaling pathway

**Figure 4:**
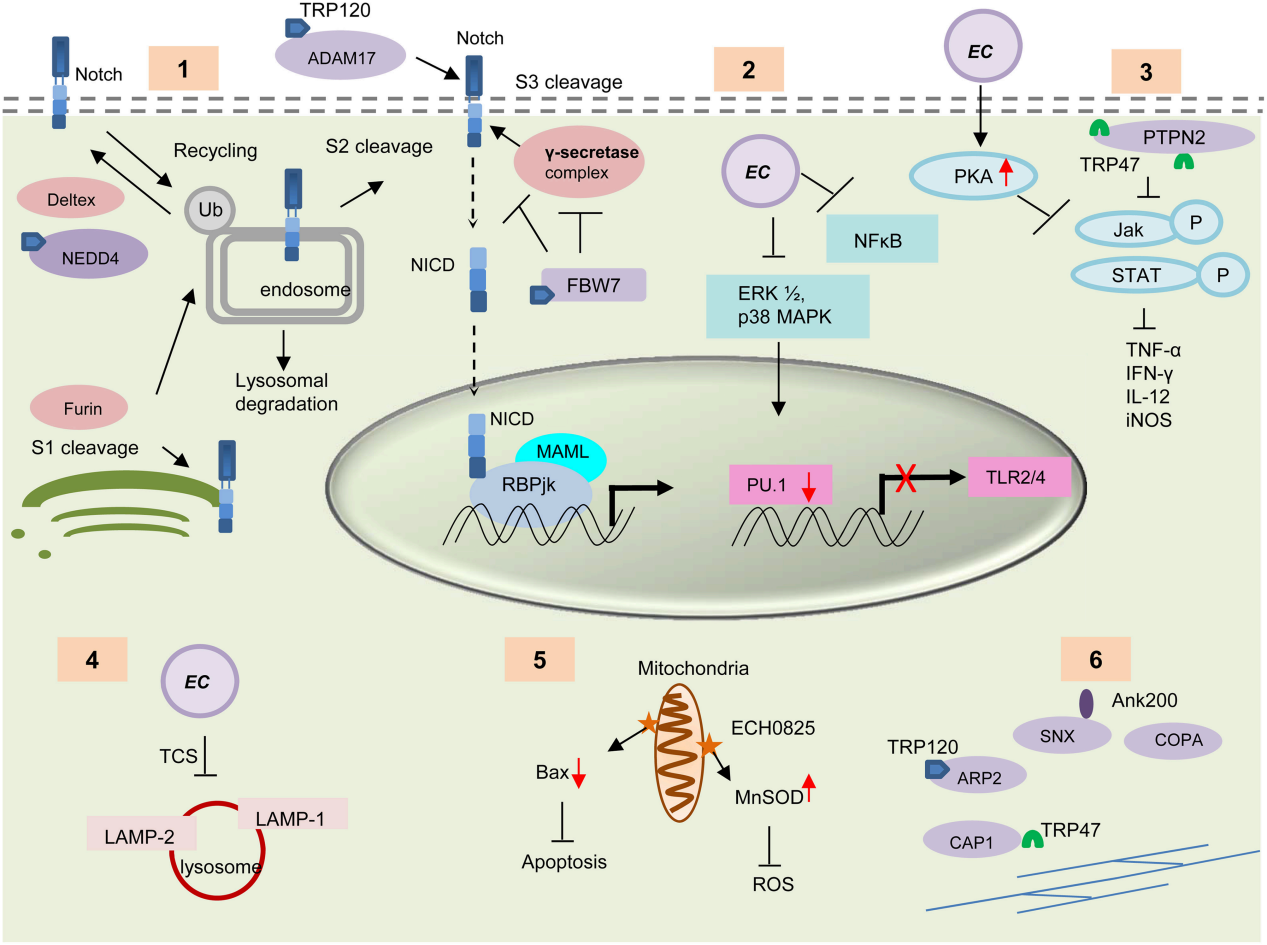
**Survival strategies used by *E. chaffeensis* during intracellular development. (1)** TRP120 interacts with some important enzymes involved in the activation and regulation of host Notch signaling pathways e.g., ADAM17, NEDD4L, and FBW7. These TRP-protein interactions might play an important role in modulation and exploitation of the Notch pathway. **(2)** *E. chaffeensis* causes decreased PRR (TLR 2 and 4) expression through downregulation of ERK1/2 and p38 MAPK cell signaling molecules and subsequent inhibition of PU.1 transcription factor. **(3)** Interaction of TRP47 with host protein PTPN2 or induction of host cell protein kinase A (PKA) by *Ehrlichia* causes inhibition of IFN-γ mediated tyrosine phosphorylation of JAk/STAT and thus causes decreased cytokine production. **(4)** *E. chaffeensis* uses a two component system (TCS) for the inhibition of phagosome lysosome fusion and thus protect itself from degradation by autophagy. **(5)** The effector protein ECH0825 protects *Ehrlichia* from ROS by induction of MnSOD and inhibits Bax mediated apoptosis. **(6)** TRPs interact with host cytoskeletal proteins to facilitate exocytosis or filopodium formation, which helps direct cell to cell transfer. TRP interacting proteins are shown in purple.

The Notch signaling is an evolutionarily conserved pathway in eukaryotes. It plays important roles in cell proliferation and differentiation, and thereby influencing cell fate (Artavanis-Tsakonas et al., 1999; Hoyne, 2003; Fortini, 2012; Radtke et al., 2013). Recently this pathway has been recognized as an important regulator of the innate and adaptive immune responses including inflammation, autophagy (Barth and Kohler, 2014), apoptosis (Palaga, 2003), Toll-like receptor (TLR) expression (Zhang et al., 2012), T and B cell development (Hoyne, 2003), and MHC class II expression (Ganta et al., 2002) in different immune cells. Cleavage of the Notch receptor by furin, ADAM metalloprotease and γ-secretase, releases the transcriptionally active intracellular domain (NICD), which translocates to the nucleus and forms a tri-protein complex with RBPjκ (CSL) and MAM to activate Notch target gene transcription (Barrick and Kopan, 2006; Kovall, 2007). Recently, TRP120 interaction with host genes associated with the Notch signaling pathway, e.g., *notch1*, was reported (Zhu et al., 2011). TRP120 interacts with ADAM17 metalloprotease, a critical enzyme involved in Notch signaling pathway, and with important regulators of Notch signaling such as NEDD4L and FBW7 (Luo et al., 2011). Both proteins act as negative regulators of Notch signaling (Figure 4). NEDD4 E3 ligase ubiquitinates Notch and regulates Notch cell surface expression by triggering its removal from the cell surface and targeting it for lysosomal degradation (Wilkin et al., 2004). Both the Notch receptor and the γ-secretase proteolytic protein component presenilin are substrates of FBW7 (Wu et al., 1998; Li et al., 2002). Modulation of Notch pathways by intracellular bacteria such as *Salmonella typhimurium, Mycobacterium bovis* and *Bacillus anthracis* has been reported where the bacteria altered the Notch pathway to regulate inflammation in the host cell (Narayana and Balaji, 2008; Larabee and Ballard, 2014). Crosstalk between Wnt and Notch signaling pathways has also been widely studied (Collu et al., 2014). The interaction of TRP effectors with critical Notch signaling components suggests that the Wnt pathway is undoubtedly not the only conserved pathway that is modulated or exploited by *Ehrlichia*. Further studies are needed to fully characterize the Notch pathway during infection and determine how the Wnt and Notch pathways are exploited to promote ehrlichial survival.

## Modulation of innate immune response

*E. chaffeensis* preferentially infects mononuclear phagocytes and has evolved remarkable strategies to survive and replicate within these primary immune cells. During infection, the immune recognition receptors and the cytokine responses which serve as the primary host defense mechanism are downregulated. *Ehrlichia* induces phagocytosis to enter the host cell, but inhibits the fusion of the *Ehrlichia* vacuole with lysosomes. It also manipulates several other host responses such as apoptosis, ROS production and IFN-γ responsiveness (Figure 4).

### Differential expression of pattern recognition receptors (PRRs)

Innate immune cells such as monocytes and macrophages express many pattern recognition receptors (PRRs) that are activated upon recognition of pathogen-associated molecular patterns (PAMPS). Different PRRs expressed on the cell surface or endosomal membrane recognize different PAMPS and activate NFκB, ERK1/2, p38 MAPK, and JNK pathways, causing increased proinflammatory cytokine and chemokine production. This response plays a critical role in pathogen elimination (Guha and Mackman, 2001; An et al., 2002). TLR2 and TLR4 are the most well characterized PRRs that detect lipoproteins and LPS, respectively (Takeuchi et al., 1999). Though *E. chaffeensis* lacks the genes required for biosynthesis of LPS and PG, this unique cell wall structure does not prevent detection by immune cells. Studies have shown that inhibition of TLR4 causes decreased levels of nitric oxide and IL-6 secretion by macrophages and results in short term persistence of *E. chaffeensis* (Ganta et al., 2002). Moreover, *in vivo* studies demonstrated that TLR2/4-dependent immune responses play a protective role in *E. chaffeensis* clearance (Chattoraj et al., 2013). However, TLR2/4 and CD14 expression and the related cytokine production are downregulated during ehrlichial infection. The underlying mechanism involves inhibition of ERK1/2, p38 MAPK that regulates expression of PU.1, a transcription factor required for TLR2 and 4 expression (Lin and Rikihisa, 2004). The intracellular PRRs, such as nucleotide-binding oligomerization domain (Nod)-like receptor proteins Nod1 and Nod2, are also differentially expressed during *E. chaffeensis* infection. Nod1 and Nod2 signals through Rip2 adaptor molecule, activating NFκB and MAPK, which results in production of immunoregulatory molecules such as chemokines and cytokines (Ogura et al., 2001; Kersse et al., 2011). Induction of the NLRs negatively regulates anti-ehrlichial protective immunity and causes increased inflammatory immune response, and thus enhances host susceptibility to *Ehrlichia* induced toxic shock (Chattoraj et al., 2013).

### Differential expression of cytokine and chemokines

Since *E. chaffeensis* does not express well-known PAMPs such as LPS, PG, pili, and flagella or capsule (Lin and Rikihisa, 2003a; Mavromatis et al., 2006), the PAMP-triggered cytokine and chemokine production appears to depend in part on the bacteria mediated modulation of host cell signaling molecules. Both MyD88 dependent and TLR dependent/independent cytokine response have been shown during ehrlichial infection. Differences between PRR signaling and cytokine production also exists between different *Ehrlichia* strains. *E. chaffeensis* Wakulla strain causes inflammatory cytokine production through MyD88, ERK, and NFκB, but not through TRIF, IL-1R1, or any TLR (Miura et al., 2011). *E. chaffeensis* Arkansas strain on the other hand inhibits protective cytokine production through inhibition of TLR2, TLR4, and CD14. The infected cells progressively become resistant to LPS stimulation and show decreased activation of ERK1/2, p38 MAPK and NFκB (Lin and Rikihisa, 2004). Microarray studies have also demonstrated inhibition of IL-12 and IL-18 expression during infection, which are important inducers of a Th1 mediated immune response (Zhang et al., 2004). Thus far, the only known protein that causes induction of MyD88 dependent inflammation is a low-molecular-weight penicillin-binding protein (Rahman et al., 2012). TRPs have shown to be associated with the regulation of different cytokine and chemokine gene expression. TRP120 acts as a nucleomodulin and causes induction of TNF-α, CCL20, CXCL11, and CCL2 gene expression, which suggests its role as transcriptional regulator of these cytokine and chemokines (Zhu et al., 2011). Ank200 binds to the promoter region of TNF-α and may induce TNF-α production (Zhu et al., 2009).

### Inhibition of autophagy

In eukaryotes, cellular degradation of cytoplasmic components is vital, since this cellular pathway removes toxic components and misfolded protein aggregates and protects them from invading pathogens and also provides nutrients through recycled degradation products. This intracellular degradation process known as autophagy is mediated by a unique double membrane organelle called an autophagosome, which engulfs and transports cytoplasmic components to the lysosome for degradation. It also serves as an innate immune response pathway that targets intracellular bacteria in the cytoplasm or in the phagosome for degradation (Klionsky et al., 2007; Shahnazari and Brumell, 2011). Though autophagy is normally induced during a bacterial infection, *Ehrlichia* appears to inhibit autophagy during infection. This is a very important immune evasion mechanism for ehrlichial survival since they reside in professional phagocytes, which are abundant in lysosomes. Recent studies have reported that ehrlichial vacuoles do not contain autophagy markers, and are not acidic (Cheng et al., 2014). Instead, *E. chaffeensis* resides in late endosome that fail to fuse with lysosomes (Cheng et al., 2014). Although no detailed studies have been conducted to understand how *Ehrlichia* inhibits autophagy, a role for the functional two component system in inhibition of phagosome lysosome fusion during ehrlichial infection has been reported. Treating the cells with the histidine kinase inhibitor closantel (two component inhibitor) prior to infection has been shown to increase co-localization between *E. chaffeensis* and lysosomal glycoprotein LAMP-1 (Cheng et al., 2006). Though autophagy can be induced or activated by several signal transduction events, the central regulator of autophagy is mTOR. During starvation conditions mTOR phosphorylates ULK1 and Atg13 and thus inhibits the initial ULK1 complex formation, which is the first step of the autophagophore formation. Both Notch and Wnt signaling play a crucial role in inhibition of autophagy through regulating the activation of the mTOR pathway and inhibiting the expression of the autophagy receptor p62 (Lapierre et al., 2011; Bailis and Pear, 2012; Petherick et al., 2013; Fu et al., 2014). It is likely that *E. chaffeensis* inhibits the fusion of this compartment with lysosomes by manipulating host cell signaling pathways to facilitate proliferation and survival. Although, activation of the Wnt and possibly Notch pathways occurs during ehrlichial infection and is required for survival, the role of these pathways in inhibition of autophagy has not been examined. Understanding the role of the Wnt and Notch pathways in induction of autophagophore formation and subsequent inhibition of its fusion with the lysosome during ehrlichial infection is currently under investigation.

### Inhibition of monocytes/macrophage activation signals

IFN-γ produced by T cells serves as one of the key regulators of both the innate and adaptive immune responses against intracellular pathogens. This macrophage-activating cytokine induces antigen presentation, phagocytosis, cytokine production, and regulates iron homeostasis, which is required for production of antimicrobial effectors including reactive oxygen species (ROS) and nitric oxides (NO) (Farrar and Schreiber, 1993; Collins, 2003, 2008). IFN-γ inhibits *E. chaffeensis* infection at early stages by inhibiting iron availability which is important for the survival of intracellular bacteria such as *Salmonella, Listeria, Mycobacteria* and *Ehrlichia* (Collins, 2003; Schaible and Kaufmann, 2004). However, IFN-γ shows no anti-ehrlichial effect when infection is established. The mechanisms involve induction of transferrin receptor expression on the surface and disruption of Janus kinase (Jak) and signal transducer and activator of transcription (Stat) signaling induced by IFN-γ. *E. chaffeensis* blocks tyrosine phosphorylation of Stat1, Jak1, and Jak2 in response to IFN-γ through raising PKA activity in THP-1 cells soon after infection (Lee and Rikihisa, 1998). TRP47 might play an important role in the inhibition of IFN-γ-induced tyrosine phosphorylation of Stat1, Jak1, and Jak2 by interacting with PTPN2 (Wakeel et al., 2009). PTPN2 also known as T cell PTP (TC-PTP), regulates phosphotyrosine levels in signal transduction pathways and targets several important host cell signaling receptors and components including CSF-1R, EGFR, PDGFR, IR, p52Shc, Stat1, Stat3, Stat5a/b, Stat6, Jak1, and Jak3. Both *in vivo* and *in vitro* data indicate that PTPN2 can also regulate cytokine signaling by regulating Jak/Stat pathway. Inhibition of PTPN2 causes Stat5 activation, increased production of IFN-γ, TNF-α, IL-12, and inducible nitric oxide synthase (iNOS). PTPN2 inhibition also results in increased tyrosine phosphorylation, enhanced activation of ERK, and may affect transcription factor PU.1 signaling (Stuible et al., 2008; Doody et al., 2009). TRP120 and Ank200 target genes of important components of the Jak-Stat pathway, e.g., Jak2, Stat1, Stat3, Stat5, and IFNR2, and thus might be involved in regulation of IFN-γ signaling during infection (Zhu et al., 2009; Luo et al., 2011).

### Downregulation of reactive oxygen species (ROS)

Reactive oxygen species produced by nicotinamide adenine dinucleotide phosphate (NADPH) oxidase is one of the major antimicrobial defense mechanisms used by the host. NADPH is a multicomponent enzyme which is composed of cytochrome b_558_ component (gp91^phox^, p22^phox^), three cytosolic subunits p67^phox^, p47^phox^, and p40^phox^ and a low molecular weight GTPase (Rac1/2 or Rap1A) (Babior, 1999; Fang, 2004). Upon invasion of pathogens, these components assemble to form a holoenzyme that produces a superoxide anion (${\text{O}}_{2}^{-}$) from the oxygen that serves as the starting material for production of different ROS such as hydrogen peroxide (H_2_O_2_), hydroxyl radicals, singlet oxygen, and oxidized halogens. *E. chaffeensis* lacks the genes required for ROS detoxification such as copper zinc superoxide dismutase (CuZnSOD), manganese superoxide dismutase (MnSOD), peroxidase, glutathione peroxidase/reductase, catalase, and OxyR/SoxRS regulons. These enzymes are utilized by many facultative intracellular bacteria. Because of the absence of these enzymes *Ehrlichia* is rendered uninfectious when exposed to H_2_O_2_ or ${\text{O}}_{2}^{-}$ (Barnewall et al., 1997). Interestingly, ehrlichiae can successfully replicate in monocytes and macrophages which are the primary producers of ROS by actively inhibiting or blocking ${\text{O}}_{2}^{-}$ generation. *Ehrlichia* mediated inhibition of superoxide generation is cell specific since it can inhibit the ROS production only in macrophages, but not in neutrophils (Lin and Rikihisa, 2007). The underlying mechanism involves degradation of the p22^phox^ unit of NADPH. This degradation does not require ubiquitination and occurs independently of intracellular signaling, but shows the involvement of iron and the interaction between *Ehrlichia* and host cell membrane proteins (Lin and Rikihisa, 2007). One of the *E. chaffeensis* two component systems CckA-CtrA regulates ehrlichial gene expression during late infection and plays a role in protecting ehrlichiae from ROS (Cheng et al., 2006).

### Inhibition of host cell apoptosis

In multicellular organisms, the number of cells is tightly regulated by cell division and programmed cell death, also known as apoptosis. It is an intrinsic immune mechanism which prevents proliferation of intracellular bacteria (Sly et al., 2003). In response to bacterial infection apoptosis is induced as an innate host immune response. It eliminates the pathogen in the early stages of infection, induces antigen presenting cells to engulf apoptotic bodies and allows antigens to be recognized by MHC molecules and thus induces a protective immune response (Elliott and Ravichandran, 2010). Spontaneous neutrophil apoptosis is delayed by stabilization of the mitochondrial membrane potential during *E. ewingii* infection (Xiong et al., 2008). *E. chaffeensis* also appears to suppress apoptosis to promote cell survival. Despite inhibition of multiple mitochondrial activities during *E. chaffeensis* infection, mitochondrial membrane potential is maintained and apoptosis inhibited (Liu et al., 2011). Cell cyclins and cyclin dependent kinase (CDK) expression are differentially regulated during infection. Apoptotic inhibitors e.g., IER3, BirC3, BCL2, and BCL related proteins such as MCL1 and BCL2A1 are induced during the infection (Zhang et al., 2004). On the other hand, apoptotic inducers such as hematopoietic cell kinase (HCK), BIK, and BNIP3L are downregulated during early infection (Zhang et al., 2004). The T4SS effector ECH0825, which is highly upregulated during exponential growth in human monocytes, localizes to mitochondria and inhibits Bax induced apoptosis. This protein also causes induction of mitochondrial manganese SOD (MnSOD) and decreases ROS level. The upregulation of MnSOD prevents ROS-mediated cellular damage and apoptosis (Liu et al., 2012). Y2H data demonstrates TRP-host protein-protein interactions may also modulate programmed cell death responses. Interaction of TRPs with apoptosis-associated proteins and their potential role as regulators of apoptosis have been discussed in detail in previous section (Section TRP-Host Protein Interactions). Further studies are needed to understand the cellular and molecular mechanisms involved in apoptosis regulation during ehrlichial infection.

## Targeting host epigenetic machinery

By altering host transcription and protein profile, *E. chaffeensis* promotes its survival and creates a replicative niche inside the host (Luo et al., 2011; Luo and McBride, 2012). These changes modulate a wide range of host cellular pathways that *E. chaffeensis* exploits for its own survival. Recent studies suggest that these changes in the host transcriptome and proteome are not only due to activation of different cell signaling pathways, but also due to direct interaction of pathogen-derived proteins with host chromatin and/or chromatin modifying proteins.

*E. chaffeensis* effector proteins such as Ank200 and TRP120 target genes involved in post-translational modification of histones, which includes histone deacetylase 1, 2, and 8 (HDAC1, 2, and 8) and SET domain containing protein which functions as DNA methyltransferase (DNMT). *E. chaffeensis* TRP120 also interacts strongly with chromatin-associated proteins, which include the histone methylase (NSD1), demethylases (KDM6B/JMJD3), protein components of the SWI/SNF chromatin remodeling complex (ARID1B), and PCGF5, a paralogous member of the polycomb group (PcG) proteins (Di Croce and Helin, 2013). PcG proteins fall into two functionally distinct protein complexes, Polycomb repressive complex (PRC) 1 and 2, and are involved in transcriptional repression of eukaryotic genes via post-translational modification of histones. The core components of the PRC1 complex include one subunit of a PCGF paralog (PCGF1, PCGF2/Mel-18, PCGF3, PCGF4/Bmi-1, PCGF5, and PCGF6), one subunit of a CBX (chromobox homolog) paralog and PHC (Polyhomeotic) paralog, and RING1 (really interesting new gene) paralogs (RING1/RING1b). RING1 is a functional E3 ubiquitin ligase, responsible for catalyzing ubiquitination of H2A at lysine 119 (H2AK119ub), while EZH (Enhancer of zest) homologs in PRC2 complex exhibits histone methyltransferase activity and produces tri-methylation of H3 at lysine 27 (H3K27me3) (Morey and Helin, 2010). The composition of the PRC1 complex is dynamic and the interaction of a particular PCGF isoform to its cognate RING protein results in recruitment of the other component of the repressive complex to its target site (Gao et al., 2012). Though there is an ambiguity in the process of PRC1 recruitment to its target location, the prevailing opinion is that it proceeds in a hierarchical fashion and requires prior nucleation of PRC2 and placement of H3K27me3 at the target location.

Polycomb group proteins were first identified in fruit flies (*Drosophila melanogaster*) as transcriptional repressors of *Hox* genes (Lewis, 1978). *Hox* genes encode Homeodomain containing transcription factors, involved in cellular differentiation and proliferation, and govern the anterior-posterior body patterning during embryo development (Sauvageau and Sauvageau, 2010). Since ehrlichial TRP proteins interact with host PCGF5 and most like to other polycomb group proteins (Wakeel et al., 2009; Luo et al., 2011), we are currently investigating the mechanism by which *E. chaffeensis* epigenetically regulates *Hox* gene expression to prolong its survival inside the host cell.

## Conclusion

Ehrlichiosis is difficult to diagnose, and delayed treatment can lead to serious complications and even death. Currently, there are no vaccines available for HME, and therapeutic options are limited. Rapid growth in antibiotic resistance among microbes and the lack of broader therapeutic options is concerning. Recent advances in our understanding of the pathogenesis of ehrlichial infection, molecular pathogen-host interactions, characterization of newly discovered TRPs and Anks and defining their role in exploiting host PTM, conserved cell signaling pathways and modulation of epigenetic machinery have provided new targets for therapeutics. Moreover, the TRPs contain species-specific epitopes that are highly immunogenic and protective, which suggests they can be used as vaccine candidates, and that the passive transfer of antibodies can serve as a therapeutic. Considerable advances have been made in understanding the cellular and molecular mechanisms used by the organism in reprogramming conserved cell signaling pathways to modulate cellular processes that enables ehrlichiae to survive inside phagocytic cells. Moreover, recent understanding of the role of these effector molecules in exploiting host PTMs and modulating host epigenetic machinery suggest their moonlighting functions in manipulating multiple host cellular processes. *E. chaffeensis* represents a model system to investigate complex pathogen-host interaction and to explore the specific cellular pathways exploited by intracellular pathogens for survival and persistence. Thus, further studies regarding the effector mechanisms and host processes that are affected by these modulations will be beneficial for designing new therapeutics for *Ehrlichia*, as well as other intracellular bacteria.

## Author contributions

TTL wrote the manuscript. TF, TL, SM, and BZ contributed to the writing of the manuscript. JWM directed and contributed to the writing of the manuscript.

### Conflict of interest statement

The authors declare that the research was conducted in the absence of any commercial or financial relationships that could be construed as a potential conflict of interest.
